# Supplementary material for: The interactome of intact mitochondria by cross-linking mass spectrometry provides evidence for coexisting respiratory supercomplexes
Source: Mol Cell Proteomics. 2017 Dec 8;17(2):216–32. doi: 10.1074/mcp.RA117.000470 (PMC5795388; doi:10.1074/mcp.RA117.000470)
Supplement: Supplemental Data [file supp_RA117.000470_133922_0_supp_23978_fzffwf.zip › spectra_annotation/mito_spectra_annotation/186-1-5-1-75-1.pdf]

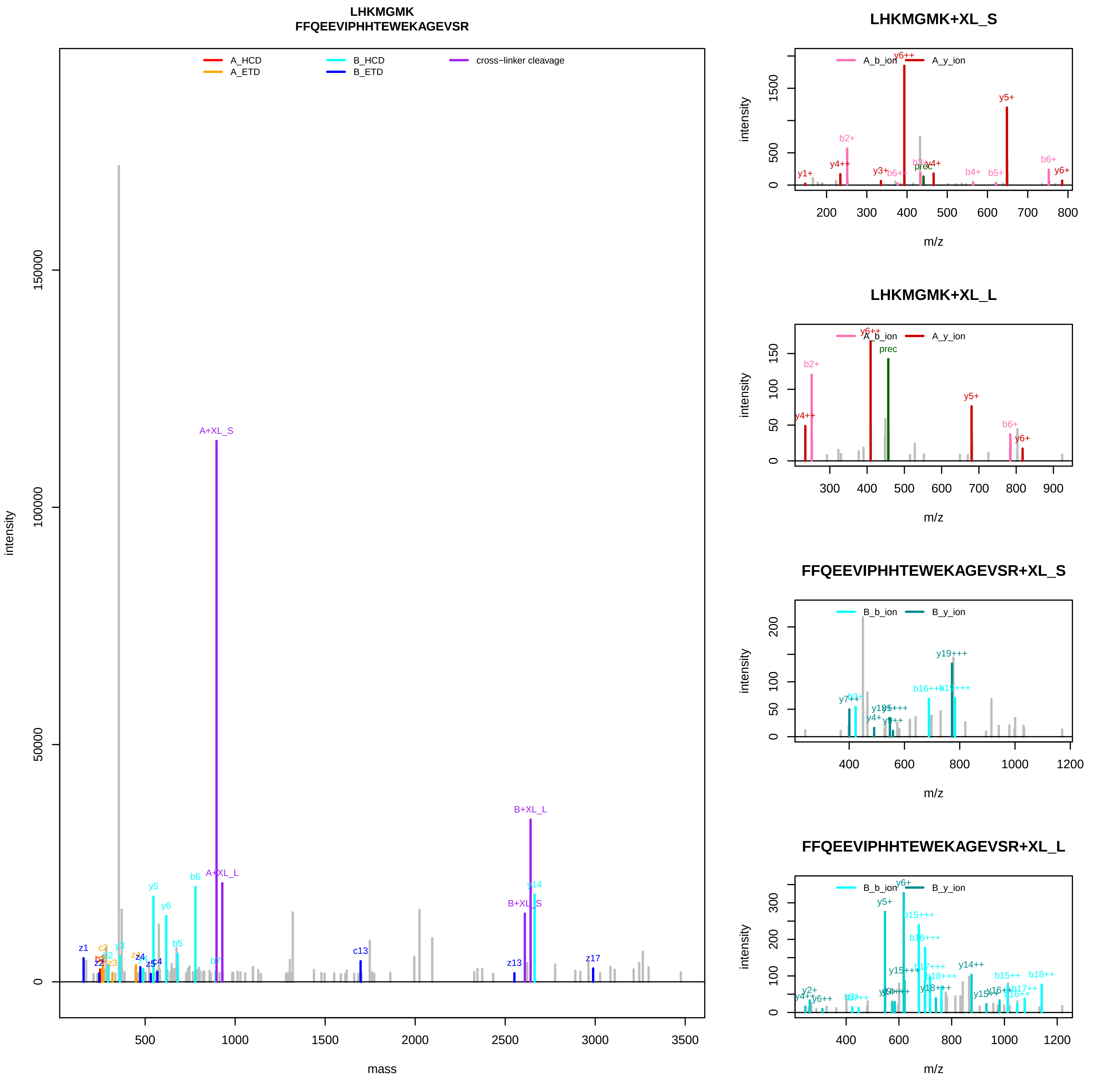
LHKMGMK
FFQEEVIPHHTEWEKAGEVSR
A_HCD
A_ETD
B_HCD
B_ETD
cross−linker cleavage
A+XL_S
A+XL_L
B+XL_L
B+XL_S
y5
y6
b6
b5
y14
c13
z13
z17
z1
intensity
mass
0
50000
100000
150000
500
1000
1500
2000
2500
3000
3500
LHKMGMK+XL_S
A_b_ion
A_y_ion
y6++
y5+
b2+
y4++
y1+
y3+
prec
b4+
b5+
b6+
y6+
m/z
LHKMGMK+XL_L
y5+
y4++
prec
FFQEEVIPHHTEWEKAGEVSR+XL_S
B_b_ion
B_y_ion
y19+++
y7++
FFQEEVIPHHTEWEKAGEVSR+XL_L
y6+
y5+
b15+++
b16+++
y14++
b15++
b18++
y2+
y6++
y15++
